# Phenolic Profile of Edible Honeysuckle Berries (Genus *Lonicera*) and Their Biological Effects

**DOI:** 10.3390/molecules17010061

**Published:** 2011-12-22

**Authors:** Tunde Jurikova, Otakar Rop, Jiri Mlcek, Jiri Sochor, Stefan Balla, Ladislav Szekeres, Alzbeta Hegedusova, Jaromir Hubalek, Vojtech Adam, Rene Kizek

**Affiliations:** 1 Faculty of Central European Studies, Institute of Natural and Informatics Sciences, Constantine the Philosopher University in Nitra, Nabrezie mladeze 91, SK-949 76 Nitra, Slovakia; 2 Department of Food Technology and Microbiology, Faculty of Technology, Tomas Bata University in Zlin, Namesti T. G. Masaryka 275, CZ-762 72 Zlin, Czech Republic; 3 Department of Chemistry and Biochemistry, Faculty of Agronomy, Mendel University in Brno, Zemedelska 1, CZ-613 00 Brno, Czech Republic; 4 Central European Institute of Technology, Brno University of Technology, Technicka 3058/10, CZ-616 00 Brno, Czech Republic; 5 Department of Chemistry, Faculty of Natural Sciences, Constantine the Philosopher University in Nitra, Tr. A. Hlinku 1, SK-949 76 Nitra, Slovakia; 6 Department of Microelectronics, Faculty of Electrical Engineering and Communication, Brno University of Technology, Technicka 10, CZ-616 00 Brno, Czech Republic

**Keywords:** edible honeysuckle, polyphenolic compounds, anthocyanins, antioxidant activity, biological effect

## Abstract

The current status of research on polyphenolic compounds in the berries of edible honeysuckle and their biological effects, including recommended utilization, are reviewed. The major classes of phenolic compounds in the blue berried honeysuckle are flavonols (quercetin, rutin, quercitrin) and flavanes (proanthocyanidins, catechins) and anthocyanins. Cyanidin-3-glucoside and cyanidin-3-rutinoside are considered as major anthocyanidins in edible honeysuckle berries. Such a high level of antioxidant activity in the berries of different species of the genus *Lonicera* is especially due to the high level of polyphenolic compounds, especially anthocyanins. These berries seem to be prospective sources of health-supporting phytochemicals that exhibit beneficial anti-adherence and chemo-protective activities, thus they may provide protection against a number of chronic conditions, e.g., cancer, diabetes mellitus, tumour growth or cardiovascular and neurodegenerative diseases.

## 1. Introduction

The human diet contains a huge variety of non-nutrient components whose implications in metabolism have beneficial health significance. The interest in the investigation of active components, especially phenolic compounds, from natural sources has greatly increased in recent years. Polyphenol compounds are widely diversified [1], these compounds are classified into different groups as a function of the number of phenol rings and of the structural elements that bind these rings to one another. Distinctions are thus made between the phenolic acids, flavonoids, stilbenes and lignans [2]. Flavonoids can be divided into six subclasses as a function of the type of heterocycle involved: flavonols, flavones, isoflavones, flavanones, anthocyanidins and flavanols (catechins and proanthocyanidins) [3].

Berries, like many other fruits, are rich in phenolic compounds and these include biphenyls, flavonoids and phenolic acids [4]. The fact that berry phenolics exhibit antioxidant properties is widely accepted [5], but certain types of phenolic compounds show greater antioxidant activity than others. Nowadays less known fruit species are also receiving much attention for their health benefit substances, including antioxidants, antimutagens and anticarcinogens, used as a prevention of various cancer and age-related diseases [6]. Although numerous types of berry fruit are consumed worldwide [7], these fruit species have been extensively studied due to their potential high antioxidant activity [8]. The less known edible honeysuckle has received attention recently as a novel berry crop for its profile of phenolic phytochemicals [9], which could be considered beneficial for consumers.

The genus *Lonicera*, which includes the above mentioned honeysuckle, is well-known as ornamental shrubs planted for their sweet scented flowers. Several species designated as “edible or blue honeysuckle” have edible, sweet-tasting fruits [10]. Different authors recognize from one to 17 species of edible honeysuckle [11,12,13]. These included botanical species with varieties such as the following most utilized ones: *Lonicera edulis*, *Lonicera kamtchatica*, *Lonicera altaica*, and *Lonicera boczkarnikovae* which originated from Russia, and *Lonicera caerule*a var*. emphyllocalyx* from Hokkaido, Japan [14]. By morphological, anatomical, biochemical and DNA analyses, as well as ploidy studies and geographical mapping of blue honeysuckle genetic resources, it has been found that genetic diversity of the crop in Eurasia is represented by four main species that are diploid endemic ones, namely *L.edulis* Turcz. ex Freyn, *L.boczkarnikowae* Plekh., *L.iliensis* Pojark and the tetraploid *L.caerulea* L. Only *L*. *caerulea* [15].

**Figure 1:**
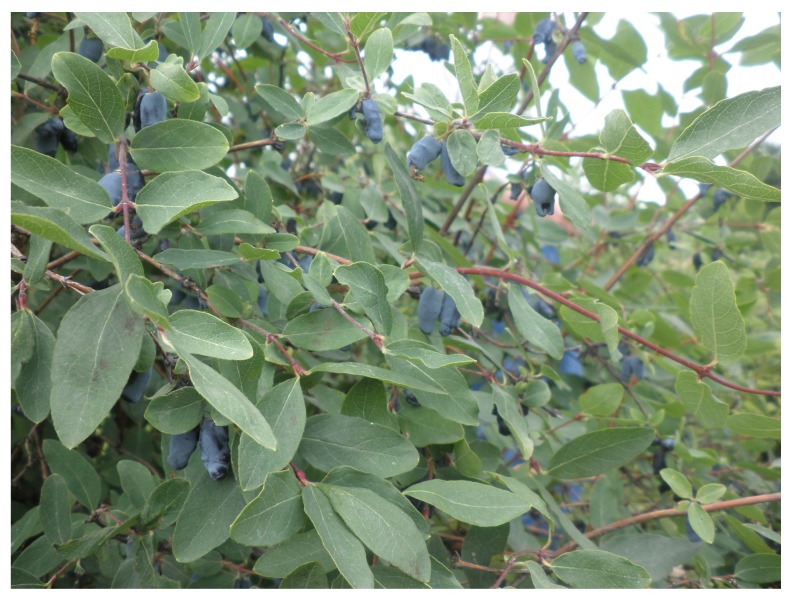
*Lonicera edulis* with fully ripened berries.

Despite the wide variety of edible honeysuckle species, tasty sweet berries with delicious aroma are characteristic only for some of them; out of these tasty varieties similar to blueberry, only crops from *Lonicera kamtschatica* and *Lonicera edulis* (Figure 1) are recommended [16], as distinct bitterness may occur in some species like *Lonicera altaica* [17] and *Lonicera pallasii* [18], which is caused by esters of malic and citric acids. Blue honeysuckle was mentioned for the first time as a horticultural plant in 1894 by the proponent of cultivating *Lonicera* in orchards, T. D. Mauritc. Since then collecting missions by the N. I. Vavilov Research Institute of Plant Industry have assembled a unique collection numbering over 500 accessions. Moreover, Hokkaido Island in Japan has a history of using blue honeysuckle that goes back hundreds of years [19].

With respect to the results of a growing number of investigations through analytical studies [13,20,21,22,23] and preliminary research of the fruit extracts, the edible honeysuckle has potential as a commercial berry crop for northern latitudes [24]. Berries are mostly elongated narrow in diameter or round shaped, have high vitamin C levels, and high antioxidant activity [25]. Among lesser-known berry crops, the edible honeysuckle (blue-berried honeysuckle) is considered to be a good source of phenolic compounds, especially anthocyanins due to their high antioxidant activity. Among other positive features we can mention are early ripening (2 weeks before strawberries), exceptional hardiness, and the factsthey are not so demanding on soil and climatic conditions (they require only a lot of moisture) and are rarely attacked by pests and diseases [26].

## 2. Polyphenolic Profile of Edible Honeysuckle

Fruits of *Lonicera altaica*, *Lonicera caerulea* and *Lonicera edulis* were reported to contain triterpenoic acids, β-carotene, ascorbic acid, anthocyanins, catechol, flavonols, chlorogenic acid and other acids [27]. Among examined samples of small berries native to Western Canada, it was found that berries of edible honeysuckle fruits contained the highest amount of polyphenolic compounds—1.11 mg of gallic acid equivalent per 100 g—Among all examined small berries [28]. The phenolic fraction from berries of *Lonicera kamtschatica*, which accounts for 4% of fresh weight, contains 3.50% phenolics, including anthocyanins (18.50%), flavonoids and phenolic acid, while the phenolic fraction of *Lonicera edulis* phenolics represents 0.40–1.50% of fresh weight [21].

The papers on chromatography of alcohol extracts of the edible honeysuckle often focus on the presence of 5–12 phenolic compounds in relation to species. Analyses of these fractions and their localization on the chromatogram make it possible to identify them as hydroxycinnamic acids, flavonols and flavons [29]. The berries of *Lonicera caerulea* have a high content of anthocyanins (1.40 mg/100 g), phenolic acids (160 mg/100 g) and flavonoids (140 mg/100 g). The other predominant compounds are represented by proanthocyanins (700 mg/100 g), catechins (650 mg/100 g) rutin (100 mg/100 g), and smaller amounts of quercetin and isoquercetin (30 mg/100 g) [23,30]. The content of polyphenolic compounds is statistically significantly influenced by species [26,31].

### 2.1. Phenolic Acids

Phenolic acid compounds seem to be universally distributed in plants, and they have been the subject of a great number of chemical, biological, agricultural, and medical studies [32]. Two classes of phenolic acids can be distinguished, depending on their structure: derivatives of benzoic acid and derivatives of cinnamic acid. They consist of benzene as a basic unit bonded to a carboxylic acid group (benzoic acids) or to propenoic acid (cinnamic acids). Both structures can be found with different hydroxylation levels [33,34]. Hydroxycinnamic acid compounds occur most frequently as simple esters with hydroxycarboxylic acids or glucose, while the hydroxybenzoic acid compounds are presented mainly in the form of glucosides. Furthermore, phenolic acids may occur as esters or glycosides conjugated with other natural compounds such as flavonoids, alcohols, hydroxyfatty acids, sterols, and glucosides in “food plants”. Moreover, hydroxycinnamic acid amides appear to be common constituents [32].

*p*-Coumaric, ferulic, caffeic and sinapic acids are widespread in fruits and vegetables. These hydroxycinnamic acids are present mainly as derivatives. The most common are esters of caffeic, coumaric and ferulic acids with D-quinin and, in addition with D-glucose. Due to the fact that quinic acid has four -OH groups, the 3- and 5- isomers are preferred, 3-, 4-, and 5-caffeoylquinic- acids are identical to neochlorogenic acid, cryptochlorogenic acid and chlorogenic acid. Isochlorogenic acid is a mixture of di-O-caffeoylquinic acids [35]. Hydroxycinnamates, especially neochlorogenic acid, and chlorogenic acid predominate in plums (*Prunus domestica*) [34], chlorogenic acid is the major hydroxycinnamic acid in apple fruit [36], chlorogenic and ferulic acid are present in the highest amount in fruits of *Citrus paradise*. In addition, caffeic, chlorogenic, *p*-coumaric and ferulic acid are typical of berries of *Vaccinium* species [37].

The hydroxybenzoic acids that are found in various fruits and occur mostly as esters include salicylic acid (2-hydroxybenzoic acid), 4-hydroxybenzoic acid, gentisic acid (2,4-dihydroxybenzoic acid), protocatechic acid (3,4-dihydroxybenzoic acid), gallic acid (3,4,5-trihydroxybenzoic acid), vanilic acid (3-methoxy-4-hydroxybenzoic acid) and ellagic acid. The hydroxybenzoic acids are derived from hydroxycinnamic acids by pathway analogous to the β-oxidation of fatty acids [35]. Sinapic, caffeic and *p*-coumaric acids are the major bound phenolic acids while *p*-coumaric, 2,4-dihydroxybenzoic and vanillic acids are the predominant free acids in cranberries [38].

Zadernowski *et al.* identified 5,418.2 ± 228 mg/100 g phenolic acids (dry weight) in blue-berried honeysuckle by gas chromatography coupled with mass spectrometry, with hydroxycinnamic acids and derivatives (61.1%), especially *p*-coumaric acid and *m*-coumaric acid, occupying a leading position [39]. Deineka *et al.* also identified the presence of chlorogenic (0.42%), caffeic (0.14%) and ferulic acid (0.10%) as the most abundant ones in the phenolic fraction of *Lonicera kamtschatica* berries, and the content of protocatechic, gentisic, rosmarinic and vanillic acids was only 0.08% in total [23]. The content of chlorogenic acid and its isomers is significantly dependent on locality of cultivation and species range in amounts from 33.10 (*Lonicera caerulea* from Karelia, Cola peninsula) up to 99.30 mg/100 g (in *Lonicera boczkarnikowae* from the Primorski territory) [40]. The different cultivation conditions do not seem to influence significantly the content of chlorogenic acid and its isomers (27.30–60.30 mg/100 g) as it was proven by Orincak *et al.*, who studied different forms of the edible honeysuckle planted in the territory of Slovakia [41].

Blue-berried honeysuckle contain an amount of hydroxycinnamic acid derivatives (30.40–156.20 mg/100 g) comparable to small berries like blueberries (114.90 mg/100 g) [42] and black currants (58–93 mg/100 g) [43] and represent a higher amount than found in plums (*Prunus domestica*) (20–50 mg/100 g) [44]. Hydroxybenzoic acids are present in a smaller amount, with a predominance of gallic acid (240 mg/kg) and 4-aminobenzoic acid (170 mg/kg) in edible honeysuckle compared to the previously mentioned species [45]. Free phenolic acids constitute only a minor portion of phenolic acids (1.70–4.20%) for all berries, bound phenolic acids are presented mainly in the form of esters (69.70%). Phenolic acids bound by glycoside linkages constitute 28.60%. All found fractions of phenolic acids are given in Table 1 [39].

**Table 1:** Phenolic acids in berries of blue honeysuckle - total amount, free and bonded as esters and glycosides (mg/kg).

Phenolic Acid	Total Amount	Free	Esters	Glycosides
gentistic acid	153.50	1.50	116.80	35.20
gallic acid	44.30	0.10	43.80	0.40
*o*- pyrocatechic acid	28.60	-	22.50	6.10
protocatechic acid	144.40	2.30	105.20	36.90
salycil acid	1234.90	9.0	824.80	401.10
vanilic acid	21.10	-	10.20	10.90
caffeic acid	598.20	22.40	536.60	39.20
*m*-coumaric acid	2014.50	6.40	1402.0	606.0
*p*-coumaric acid	987.10	23.50	631.70	331.90
dimetoxycinnamic acid	44.20	-	29.90	14.30
ferulic acid	36.90	20.70	13.10	3.10
hydroxycaffeic acid	51.90	-	-	46.50
*p*-hydroxyvinear acid	10.30	0.90	9.40	-
*p*-hydroxylactic acid	48.30	0.50	29.20	18.60
chlorogenic acid	120.50–166.0	-	-	-

### 2.2. Flavonoids

#### 2.2.1. Flavonols, Flavons and Flavanols

Flavonoids are a group of polyphenolic compounds widely distributed throughout the plant kingdom that inhibit the oxidation chain initiation and prevent chain propagation by acting as free radical scavengers [46]. The content of flavonoids in the berries of edible honeysuckle is statistically significantly dependent on species—in the berries of *Lonicera kamtschatica* the content found is 1,900 mg/100g, and in *Lonicera edulis* 600–1,800 mg/100 g [47].

Flavonols are usually found as their glycoside derivatives in plants and as with other flavonoids, various mono and disaccharides are a part of the flavonol structure. Flavonols are the most ubiquitous flavonoids in fruit, and quercetin and kaempferol are the main representatives [44]. More than 1,400 glycosylated flavonols and glycosides of quercetin, such as quercitrin and rutin have been found to present an *ortho* 3,4-dihydroxy moiety in the B- ring (e.g., catechin and quercetin) and 3,3-double bond in combination with the 4-keto group and 3-hydroxyl group in the C-ring for effective electron delocalization (e.g., in quercetin) as long as the *o*-dihydroxy structure is also present [48,49].

Within the blue honeysuckle Plekhanova *et al.* investigated the presence of free flavonols—Rutin, quercetin and isoquercetin (presented in berries of all studied edible honeysuckle from the territory of Slovakia in the amount of 8.2–10.1 mg/100 gtogether with diosmin [29]). A reliably high content of rutin in the amount from 27 to 48 mg/100 g has been recorded in *Lonicera iliensis* and *Lonicera boczkarnikowae* berries, also in the different forms of *Lonicera kamtschatica* in the samples from Slovakia (13–41 mg/100 g). Moreover, quercetin in amounts over 10 mg/100 g was present only in *Lonicera edulis* and it was found in trace amounts in different forms of *Lonicera caerulea* (1.3–7.0 mg/100 g) [41].

In the berries of edible honeysuckle Petrova determined 47 mg/100 g of glucosides of flavonols such as luteolin-7-glucoside (8.2–10.3 mg/100 g), luteolin-7-ramnoglucoside and 5,3-dioxy-4-methoxyrutinoside [42]. Sapiro examined the differences between species regarding their flavonol content (calculated as quercetin)—*Lonicera edulis* berries (142 mg/100 g of fresh weight), *Lonicera altaica* (74.4 mg/100 g) and *Lonicera caerulea* (122 mg/100 g). The differences were also reflected in the content of catechins—*Lonicera edulis* (344.5 mg/100 g), *Lonicera altaica* (292.5 mg/100 g) and *Lonicera caerulea* (298.3 mg/100 g) [50].

The amount of flavonols in blue honeysuckle fruits (12.60–32.80 mg/100 g) was lower than in bilberries (54–100 mg/100 g), black currants (72–74 mg/100 g) and blueberries (60.9 mg/100 g) [51]. The major class of phenolic compounds in the blue berried honeysuckle are flavanols, which exist in both monomer form (catechins) and polymer form (proanthocyanidins) [44]. The leading position among the berries of the edible honeysuckle is occupied by proanthocyanidins. It was found that in Russia the content of this compound ranged from 195 to 772 mg/100 g [29], with similar ranges seen in Slovakia (470–616 mg/100 g) [41]. *Lonicera boczkarnikowae* and *Lonicera iliensis* differ from other species by their reliably high and low content, respectively. Among catechins, monomeric forms predominate, ranging from 122 to 625 mg/100 g [40]. In the samples originating from Slovakia these values are lower (280–413 mg/100 g) [41]. A lower content of catechins in berries is characteristic of the diploid species of *Lonicera edulis* and *Lonicera iliensis* while an increased one has been noticed for *Lonicera caerulea.* The presence of flavanes, flavonols and flavones depends on species and geographic origin and it is given in Table 2 according to results in the literature [29,41].

**Table 2:** Content of flavonoids—flavanes, flavonols and flavones in berries of different *Lonicera* species (mg/100 g).

Species	Geographicorigin	Proantho-cyanidins	Freecatechins	Flavonols	Rutin	Isoquer-cetin	Quercetin	7-0-Luteolinglucoside
*L. caerulea*	Kamtschatka	400	244	–	7.20	9.10	2.80	11.60
	Kurily islands	252	225	–	8.80	8.80	0	10.00
	Primorski territory	694	625	–	16.60	7.20	0	9.50
	East Syan mountains	664	528	–	0	7.0	1.0	10.20
	Kyrghyzstan	536	322	–	8.0	11.10	2.80	6.0
	Karelia	423	302	–	11.0	4.10	0	6.50
	Far East	–	298.3	122	–	–	–	–
*L. edulis*	Transbakalia	436	185	–	11.60	11.90	10.50	0
	Far East	–	344.5	139	–	–	–	–
*L. boczkarnikowae*	Primorski territory	772	429	–	27.40	10.80	0	0
*L. iliensis*	Kazachstan	195	122	–	48.60	8.90	0	13.60
*L. villosa*	Canada	232	240	–	7.70	6.20	0	4.70
*L. altaica*	Far East	–	292.50	74.0	–	–	–	–

#### 2.2.2. Anthocyanins

Anthocyanins and leukoanthocyanins represent the major class of polyphenolic compounds in blue honeysuckle berries [51]. The content of anthocyanins significantly depends on species and different conditions of cultivation and presents values from 400–1,500 mg/100 g. The highest amount of anthocyanins as 3,490 mg/100 g of fresh weight was reported in berries of *Lonicera altaica* from Altaj [47]. They are present in a lower amount in *Lonicera edulis* berries (1,207.5 mg/100 g), *Lonicera Turczaninowii* (1,452.5 mg/100 g) and *Lonicera caerulea* (1,470 mg/100 g; the values are calculated for cyanidin-3-glucoside). Cyanidin-3-glucoside dominates in berries of most *Lonicera* species [52], however, this is the most common anthocyanin distributed in Nature [53]. Aside from cyanidin-3-glucoside Gazdik *et al.* also reported cyanidin-3-rutinoside as a predominant anthocyanin in berries of *Lonicera caerulea* [54]. In addition, Terahara *et al.* also identified cyanidin-3,5-diglucoside as a major anthocyanin according to HPLC analyses of *Lonicera caerulea* berries. In addition, the presence of malvidin-3-glucoside and cyanidin-3-gentiobiosid were found [55]. 

Chaovanakilit *et al.* examined the variation in the proportion of anthocyanidins as follows: cyanidin-3-glucoside (79–88%) as a major anthocyanidin, cyanidin-3-rutinoside (1–11%), cyanidin-3,5-diglucoside (2.2–6.4%). The mentioned authors reported for the first time the presence of peonidin 3-glucoside (2.8–4.5%), peonidin-3-glucoside (0.3–1.3%) and pelargonidin-3-glucoside (0.2–1%) [15]. Mariassyova *et al.* studied samples of *Lonicera edulis* originating from Slovakia and determined the ratio of cyanidin-3-glucoside, cyanidin-3,5-diglucoside and peonidin-3,5-diglucoside as 89:4:7 [56]. According to Caganova the major anthocyanidins in berries of *Lonicera caerulea* are glucosides and rutinosides of cyanidin, peonidin, delphidin and pelargonidin [57]. Bakowska *et al.* also reported the presence of petunidin and malvidin [58]. The fruit of *Lonicera caerulea* did not contain acetylated anthocyanins [23]. 

Anthocyanins are found mainly in the skin of fruits, except for certain berries like cherries and strawberries [44]. Anthocyanins extracted from berries of blue honeysuckle also achieve a higher concentration in skin as 12.28 g/kg than in the flesh as 4.34 g/L. The yield of anthocyanins from *Lonicera edulis* berries is higher (in skin 9.25–17.7 g/kg, in flesh-saft 1.71–9.68g/L) in comparison with *Lonicera kamtschatica* (in pomace 8.72–15.99 g/kg, saft 0.61–6.32 g/L) [16]. The content of anthocyanins in honeysuckle berries is comparable with the elderberry (*Sambucus nigra*, cultivar “Hasberg”) and chokeberry (*Aronia melanocarpa*, cultivar “Nero”) [59] and represents the richest and most important source (8.58–19.18 g/kg) among studied lesser-known species such as black mulberries, cornelian cherries, blackberries, blackthorn and rowanberries [60,61]. There are no statistically significant differences in anthocyanin content among studied lesser known fruit species—*Lonicera kamtschatica* (5.67 g/kg), chokeberry—*Aronia melanocarpa* (5.12 g/kg), blueberry—*Vaccinium corymbosum* (4.16 g/kg) [62]. Fuzzy cluster analyses proved that species displayed a more significant effect in the anthocyanin content in comparison with the effect of locality [16], which is contrary to the results of a study by Plekhanova who found no statistically significant differences between *Lonicera kamtschatica* and *Lonicera edulis* in terms of polyphenolic compounds [17]. In the case of *Lonicera kamtschatica* berries, during two weeks long ripening an increase in the content of anthocyanins from 1.08 to 10.38 g/kg was found [44]. 

The anthocyanin content in *Lonicera kamtschatica* berries was also affected by storage, while the accumulation of anthocyanins after 6-month storage by freezing was higher by 8.78–19.8% in the majority of the samples of *Lonicera kamtschatica* [63]. Bryksin found a decrease in anthocyanins content by 4.5–61.29% in samples of *Lonicera edulis* due to storage of the fruits [64]. According to Baloghova *et al.*, all studied samples of lesser known fruit species maintained their contents of anthocyanins, ascorbic acid and and antiradical activity after freezing down to −18 °C, thus this temperature is the most suitable for preservation of these samples [62]. The anthocyanin content is also influenced by the extraction procedure according to Gazdik *et al.*, and the most suitable one to obtain the highest yield of anthocyanins was the use of acidified methanol as the extraction medium. Comparable total anthocyanin content was obtained using a mixture of methanol and acetone [54]. 

#### 2.2.3. Stability of Anthocyanins

The extraction from fruits of edible honeysuckle provides a natural red coloured food pigment that has shown good results in trials and proved promising for the confectionary industry [12]. So far, anthocyanins have not been broadly used in foods and beverages, because they are not as stable as synthetic dyes. In fact, the colour stability of anthocyanins depends on a combination of various factors, such as the structure and the concentration of the anthocyanin, pH, temperature and the presence of complexing agents (phenols, metals, *etc*.) [65].

Gazdik *et al.*, who studied 21 clones of *Lonicera kamtschatica*, pointed to a statistically significant albeit positive weak correlation between anthocyanin and ascorbic acid content in samples studied in 2008 [45]. Jurikova *et al.* found a statistically significant positive strong correlation in the same samples in 2009 [63]. On the other hand, Plekhanova and Streltsyna recorded that a content of ascorbic acid higher than 70 mg/100g is associated with a lower accumulation of phenolics [11]. The correlation between anthocyanins and organic acids is also positive but weaker than in case of anthocyanins—ascorbic acid. A weak positive correlation also exists between the content of anthocyanins and saccharides (r = 0.027) [63].

Sunlight degrades cyanidin-3-glucoside isolated from berries of *Lonicera kamtschatica*. The loss after three months of storage in the dark was about 54% lower than after storage in the sun. UV light and pH also have strong degradative effect. Cyanidin-3-glucoside is more stable at pH 1–2, where cyanidin exists essentially in its flavylium form. At pH 3, there is an important colour loss of cyanidin due to the production of a colourless carbinol pseudobase. At pH 4–6, cyanidin is practically colourless. In this range quinoidal bases are formed and the amount of carbinol pseudobase increases [58]. Recent investigation has suggested that the molecular copigmentation of anthocyanins with other compounds (copigments) is the main colour-stabilizing mechanism in plants [66]. The complexation of copigment with anthocyanin causes a hyperchromic effect (an increase in colour intensity) and bathochromic shift (the shift of wavelength of maximum absorbance) [67].

Bakowska *et al.* studied how to stabilize cyanidin-3-glucoside isolated from *Lonicera kamtschatica.* The results showed that all investigated copigments -quercetin-5′-sulphonic acid (QSA), sodium salt of morin-5´-sulphoacid (NaMSA), rutin, quercetin, chlorogenic, tannin acid and unknown flavonoids prepared from roots of *Scutellaria baicalensis*—Enhanced the colour stability and intensity of cyanidin-3-glucoside. The maximum copigmentation effect was observed at pH 3.5. The degradation influence of UV radiation on the complex of cyanidin with copigment was stronger than heating at 80 °C. The highest colour stability effect was observed with flavones of *Scutellaria baicalensis* after heating, UV irradiation and storage. The copigmentation process was affected by pH and temperature. The temperature appears to be a basic parameter of thermodynamic feasibility of the process, because it is an exothermic and spontaneous process [58].

Zhao recorded that pigment resin from berries of *Lonicera edulis* extracted with 50% ethanol has a high absorption capacity and after repeated use (20 times) its absorption rate decreases by only 1.29%, *i.e.*, the pigment has good stability under acidic conditions. The natural pigment showed better stability at 70 °C and under acid conditions, and poor antioxidant capacity in the presence of Cu^2+^ and Al^3+^ ions. Ca^2+^, Zn^2+^ and Mg^2+^ had a positive effect on pigment stability, and Fe^3+^ has damaging effects on the pigment. Moreover, it was found that sugars, NaCl, citric acid and sodium benzoate have no impact on *Lonicera edulis* berry pigment and it can be widely used in the food, medicine and cosmetics industries as a natural plant pigment [68]. The pigment extracted from *Lonicera caerulea* berries is not stable under light, H_2_O_2_, ascorbic acid, Sn^2+^, Fe^3+^ and Cu^2+^, because these compounds greatly reduce pigment stability. Occurrence of flavonoids and polyphenols strengthens the pigment stability by the effect of increasing the concentration [69].

#### 2.2.4. Antioxidant Activity

It is well known that various berries and fruit types of less common fruit species are known to contain a high level of antioxidants. The determination of antioxidant activity is one of the possibilities for expressing the biological value of fruit, concurrently with the assessment of the main bioactive components represented in the berries. Such a high level of antioxidant capacity in the berries of different species of the genus *Lonicera* is especially due to the high level of polyphenolic compounds [13].

Anthocyanins have been shown to be strong antioxidants, and may exert a wide range of health benefits through antioxidant or other mechanisms. The correlation between anthocyanins content and antioxidant activities in fruits of chokeberry—*Aronia melanocarpa*, honeyberry—*Lonicera kamtschatica* and blackthorn—*Prunus spinosa* was studied [70]. Lipid oxidation in the liposome membrane, induced by UV-radiation, was evaluated by thiobarbituric acid reactive substances assay. The antioxidant efficiency of the studied compounds followed the order: *Aronia melanocarpa* > *Prunus spinosa* > *Lonicera kamtschatica*. Similarly, honeysuckle berry (*Lonicera kamtschatica*) among studied lesser known fruit species (black mulberries—*Morus nigra*, cornelian cherries—*Cornus mas*, blackberries—*Rubus fruticosus*, blackthorn—*Prunus spinosa*, rowanberries—*Sorbus aucuparia*) despite the highest content of anthocyanins reached up the lowest values of antioxidant activity [61]. Therefore, one may suppose that other flavonoids can also significantly contribute to the radical species scavenging activity of edible honeysuckle. Also study of Gazdik *et al.* supported the fact that phenolic acids and flavonoids mostly contributed to antioxidant activity of *Lonicera edulis* [54].

Thompson and Chaovanalikit measured the total antioxidant capacity in 11 fruit samples of different subspecies of *Lonicera caerulea* as oxygen radical absorbing capacity (ORAC) with values of 18 to 104 μmol Trolox equivalent per g of fresh weight and ferric reducing antioxidant power (FRAP) with values of 37 to 113 μmol Trolox equivalent per g of fresh weight. According to the mentioned authors the antioxidant capacity was correlated with both anthocyanin and total phenolic contents [71]. In this way anthocyanin pigments are very effective scavengers of free radicals [72]. ORAC and FRAP values were also highly correlated with hydroxycinnamic acid derivatives (r = 0.828) and flavonols (r = 0.83). However, these correlations were lower than the correlation of ORAC and FRAP with anthocyanins (r = 0.95) and total phenolics (r = 0.97), which were similar to those seen in the studies of Chaovanalikit *et al.* [15]. Although the content of hydroxycinnamic acid derivatives and flavonols of blue honeysuckle was totally different from the other berries, blue honeysuckle provided comparable ORAC (1,840–10,370 μmol of Trolox equivalent/100 g with berries of several genera: blackberries (1,300–14,600 μmol of Trolox equivalent/100 g) and black currants (1,700–11,600 μmol of Trolox equivalent/100 g) [51]. 

The high antioxidant activity of berries is maintained despite totally different cultivation conditions. In this way *Lonicera caerulea* fruits introduced into the new climatic conditions of Romania proved that berry plants provide a high content of antioxidants (12,385.63 μmol TE/mg of dry fruit for aqueous extract; 12,455.27 μmol TE/mg of dry fruit for alcoholic extract and 3,918.05 μmol TE/mg of dry fruit for total extract). The results were expressed in Trolox equivalent radical scavenging activity on mg of dry base [73]. Another study by Rop *et al.* using the DPPH (2,2-diphenyl-1-picrylhydrazyl) test in particular cultivars of *Lonicera kamtschatica* introduced into the conditions of the Czech Republic determined a high antioxidant activity, which ranged from 6.59–10.17 g of ascorbic acid equivalent/kg of fresh mass [13]. The phenolic fraction of the fruit of *Lonicera caerule*a L. originating from the Czech Republic displayed Folin-Ciocalteu reagent reducing (335.00 ± 15.00 μg of gallic acid equivalent/mg) and DPPH and superoxide scavenging activity [IC(50) 12.10 ± 0.10 and 115.50 ± 6.40 μg/mL] and inhibited rat liver microsome peroxidation [IC(50) 160.00 ± 20.00 μg/mL] [74].

## 3. Utilization and Health Benefits of Edible Honeysuckle Berries

The berries of edible honeysuckle have been widely harvested and used in folk medicine in northern Russia, China and Japan since ancient times. Their fruits, flowers, leaves, branches and bark were used in the folk medicine in the countries of their origin (e.g., branch infusion as a diuretic remedy, fresh fruits and fruit juice as a general strengthening means and they were also recommended for the treatment of some diseases of the stomach and tonsilitis for its antiseptic effect, leaf infusion for the treatment of throat and eyes, *etc.*) [75]. However, although various parts of *Lonicera* shrubs have been utilized in folk medicine for many decades, it is only recently that several phenolic matrix constituents have been suggested as the main components responsible for the health benefits of the edible honeysuckle. Nowadays it is well-known that the fruits are rich in phenolic acids, flavonoids, anthocyanins and proanthocyanidins. These compounds have been recognized to possess a wide range of biological activities such as antimicrobial, anticancer and anti-inflammatory. Because a full discussion of biological activity of polyphenols would be prohibitively long, this review will focus on main areas of implications based on main areas of studies in fruits of edible honeysuckle. The berries of the edible honeysuckle have a lot of useful therapeutic properties that have been tested on laboratory animals represented by mice and rats [63].

All observed protective effects of berries of edible honeysuckle are linked with their antioxidant properties, mainly anthocyanin content [76]. Peonidin-3-glucoside and cyanidin-3-glucoside also gave very good results in the regulation and reduction of the expression of metaloproteinases (MMPs) and urokinasesurokinasis (u-PA), which are responsible for the initiation and proliferation of metastasis among cells [67]. In this way the edible honeysuckle phenolic fraction (18.5% of anthocyanins) has perspectives in the prevention of some oxidative stress-associated diseases. Palikova *et al.* tested the effect of a berry extract on cell viability and against oxidative damage in low density lipoproteins (oxLDL), in rat microsomes and in primary cultures of rat hepatocytes and in human umbilical vein endothelial cells. The phenolic fraction inhibited rat liver microsome peroxidation, induced by *tert*-butylhydroperoxide (tBH), with IC(50) values of 160 ± 20 μg/mL. The fraction at 0.5, 1.0, and 2.0 μg/mL delayed LDL oxidation, induced by Cu^2+^, by 130 ± 20%, 200 ± 30%, and 400 ± 10%, respectively [74]. 

Gruia *et al.* showed that tumour grafting induced oxidative stress. *Lonicera caerulea* berry extracts, but only when the extract administration was started concomitantly with the tumour grafting, showed good protection against tumour grafting and a negative effect on tumour progression. Continuous administration of berry extracts starting three weeks before a tumour was grafted offered only limited protection [8]. *Lonicera caerulea* compounds block mutagenesis and have been shown to modify the process of uncontrolled cell proliferation and apoptosis *in vitro* [78].

Zheng *et al.* provided a histochemical study of the protective effects of *Lonicera edulis* berries on mouse liver damaged by carbon tetrachloride. Mice were randomly divided into three groups and examined for neutral fat by SDH (succinate dehydrogenase activity). In an experimental group a reduction of fat drops and increased succinate dehydrogenase activity was found. Mice with liver injuries induced by carbon tetrachloride also showed lower serum aspartate aminotransferase vitality and a decrease in a number of lysosomes with reduction of the activity of acid phosphatase. Based on these results it can be concluded that edible honeysuckle berries can play an important role in the treatment of metabolism disorders [78,79].

The medicinal values of fruits have long been appreciated for their therapeutic effect on cardiovascular diseases, because they are known to reduce blood pressure and there are claims of curative effects for malaria and gastrointestinal diseases [11]. Guang *et al.* studied the therapeutic effects of *Lonicera edulis* alcoholic extract on adjusting blood lipid levels in Wistar rats with hyperlipidemia as animal models. They found out after 28 days of treatment with this extract that triglyceride esters, cholesterol level and high density lipoprotein levels decreased and there were significant differences in high fat compared with a control group [80]. In addition, *Lonicera edulis* ethyl acetate extract showed a significant protective and inhibitory effect in animal experiments using mice with flooding stress ulcers. Compared with the control group, the drug treated rats had significantly increased NO (nitric oxide) and NOS (nitric oxide synthetase) in stomach (P < 0.01). In gastric tissue this effect was significantly lower. Thus, it is probable that *Lonicera edulis* ethyl acetate extract improves the healing of the ulcer mucosa [81,82]. Based on the promising results from these studies ochnaflavone was prepared, which is a medicinal herbal product isolated from *Lonicera japonica* that inhibits cyclooxygenase-2 (COX-2) dependent phases of prostaglandin D2 (PGD2) generation in bone marrow-derived mast cells (BMMC) [83].

The phenolic fraction of L. caerulea fruit may be beneficial for the adjunctive treatment of periodontitis as an agent for attenuation of the inflammatory process [74]. The polyphenolic fraction of L. caerulea (PFLC) fruit was also able to reduce most studied alterations induced by lipopolysaccharide (LPS) in gingival fibroblasts, particularly markers related to oxidative stress and inflammation. Zdarilova *et al.* found that application of PFLC (10–50 μg/mL) reduced reactive oxygen species (ROS) production, intracellular glutathione (GSH) depletion as well as lipid peroxidation in LPS-treated cells. PFLC treatment also inhibited LPS-induced up-regulation of interleukin-1β (IL-1β), interleukin-6 (IL-6) and tumour necrosis factor-α (TNF-α) and it suppressed expression of cyclooxygenase-2 (COX-2) [84]. In addition to these findings, a recent study has also shown that the freeze-dried fruit of L. caerulea and its phenolic fraction was able to reduce the biofilm formation and adhesion to the artificial surface of some bacteria such as E. coli and *Staphylococcus epidermis*. 

High antioxidant activity in edible honeysuckle berries inhibits the growth of Gram negative bacterial strains of *Eschericchia coli* and *Salmonella enterica*, and there a statistically significant linear relationship between total phenolic content and antibacterial activity against both examined bacterial strains has been proven [45]. An extract of *Lonicera caerulea* also showed a significant effect on endotoxin-induced uveitis in rats. The possible mechanism for this effect may depend especially on the ability to inhibit activation of NF-κB and the subsequent production of proinflammatory mediators such as TNF-α, prostaglandin (PG)-E2 and nitric oxide (NO). To further clarify the anti-inflammatory effects, a mouse macrophage cell line was stimulated with LPS (injection of lipopolysaccharide) in the presence or absence of blue honeysuckle extract. The treatment with this extract significantly reduced the inflammatory cell infiltration, the protein concentration, the levels of NO, TNF-α and PGE2 in the aqueous humour and improved histologic status of the ocular tissue. Major phenolic compounds responsible for this effect are cyanidin-3-glucoside (C3G), cyanidin-3-rutinoside (C3R) and chlorogenic acid (CA) [83].

Phenolic compounds in the berries of edible honeysuckle play an important role in a wide range of physiological processes, including skin-protective effects and protection against harmful UV radiation in plants. Some components of *L. caerulea* fruits such as quercetin or cyanidin-3-glucoside have been shown to protect UVA-induced damage *in vitro* and *in vivo*. The results of experiments of Svobodova *et al.* suggest that the phenolic fraction of *L. caerulea* (PFLC) and *V. myrtillus* fruits suppresses UVB-caused injury to keratinocytes. Pre- and post-treatment with PFLC significantly supressed UVA—induced ROS production, which was also revealed as a decrease in intracellular lipid peroxidation and elevation of GSH. The protection was concentration dependent, with a maximum at 50 mg/L [85]. Together with another lesser-known fruit species like cranberry (*Viburnum opulus* var. edule) the edible honeysuckle could become a promising crop plant in human nutrition [86].

## 4. Conclusions

In recent years, there has been an increasing interest in investigating polyphenols from botanical sources for their possible neuroprotective effects against neurodegenerative diseases. Gallic acid, *p*-benzoic acid, together with rutin, quercetin and quercitrin, *etc.* have strong antioxidant properties, including mobility in the bloodstream and ability to penetrate through the hematoencefalic barrier. This way quercetin seems to be the most powerful flavonoid for protecting the body against reactive oxygen species. The use of blue honeysuckle as a source of natural antioxidants, natural colorants, and an ingredient of functional foods seems to be promising. It also represents a useful addition to the prevention of a number of chronic conditions, e.g., cancer, diabetes mellitus, tumour growth, and cardiovascular and neurodegenerative diseases. Moreover, new methods and approaches how to evaluate beneficial effects of fruits extracts are still being suggested [87,88,89,90,91].
